# Is There any Additional Benefit of Multiple Doses of Tocilizumab in COVID-19 Patients?

**DOI:** 10.7759/cureus.12214

**Published:** 2020-12-22

**Authors:** Mohsin S Mughal, Ikwinder Kaur, Mili Kakadia, Chang Wang, Reem Alhashemi, Rafah Salloum, Anthony Ricca, Kenneth Granet

**Affiliations:** 1 Internal Medicine, Monmouth Medical Center, Long Branch, USA; 2 Biostatistics and Epidemiology, Rutgers University, New Brunswick, USA; 3 Rheumatology, Monmouth Medical Center, Long Branch, USA

**Keywords:** il-6 receptor inhibitors, mortality, covid-19, tocilizumab

## Abstract

Many patients with coronavirus disease 2019 (COVID-19) have a hyperactive immune response (cytokine storm) which has been incriminated in multiorgan dysfunction (MOD). Interleukin-6 (IL-6) and granulocyte-macrophage colony-stimulating factor (GM-CSF) are the key cytokines involved in mediating systemic inflammation and triggering endothelial dysfunction. To limit these effects, IL-6 receptor inhibitors (IL6ri) have been used in COVID-19 patients. The best approach regarding the total number of doses in COVID-19 patients is still unclear. In this single-center retrospective study, we investigated if multiple doses of tocilizumab (TCZ) prevented deterioration of COVID-19 patients. Patients were divided into two cohorts based on the number of TCZ doses; cohort 1 (received one dose) and cohort 2 (received ≥ two doses). In both cohorts, all-cause-mortality was the primary outcome. Of 270 hospitalized patients with COVID-19, 81 patients received TCZ. Fifty patients received one dose of TCZ and 31 received ≥ two doses. All-cause-mortality in cohort 2 remained higher (41.9%) suggesting that there was no additional benefit of multiple doses of TCZ to prevent the primary outcome. In addition, multiple doses of TCZ did not change any other secondary outcome [(ICU admission, acute kidney injury (AKI), acute respiratory distress syndrome (ARDS), acute cardiac injury (ACI), thrombotic events, septic shock, and total hospital stay].

## Introduction

Many patients with coronavirus disease 2019 (COVID-19) have a hyperactive immune response (cytokine storm) which has been incriminated in multiorgan dysfunction (MOD). These dysfunctions include acute respiratory distress syndrome (ARDS), acute renal failure (ARF), acute cardiac injury (ACI), and complications related to hypercoagulability and thrombosis. Interleukin-6 (IL-6) and granulocyte-macrophage colony-stimulating factor (GM-CSF) are the key cytokines involved in mediating systemic inflammation and triggering endothelial dysfunction which precipitates these events [1]. To limit these effects, IL-6 receptor inhibitors (IL6ri) have been used in COVID-19 patients. The use of IL6ri is being investigated through ongoing randomized clinical trials (RCTs) to establish its effectiveness [2]. Data regarding optimal timing and dosing of intervention to target cytokine release pathways with IL6ri is still evolving. In a retrospective study the patients who received IL-6ri earlier (Stage IIB, requiring Fi02<45%) were less likely to be intubated and had an increased rate of discharge from the hospital [3-4]. Tocilizumab is an IL6ri that has been used for CAR-T cell therapy associated cytokine release syndrome and the optimal dose is 8 mg/kg for the patients; however, multiple doses have been used with at least an eight-hour interval between consecutive doses based on the clinical status of patients [5]. The best approach regarding the total number of doses in COVID-19 patients is still unclear. In this single-center retrospective study, we investigated if multiple doses of TCZ prevented deterioration of COVID-19 patients.

## Materials and methods

Adult patients with confirmed SARS-CoV-2 infection by nasopharyngeal (NP) polymerase chain reaction (PCR) who were hospitalized from March 1 to August 20, 2020, were reviewed. Out of 270 patients, 81 received tocilizumab (TCZ) (IL6ri). Patients were divided into two cohorts based on the number of TCZ doses; cohort 1 (received one dose) and cohort 2 (received ≥ two doses). Both TCZ doses were weight-based (400-800 mg). Data were extracted using the hospital electronic medical record retrospectively. Categorical variables were compared by conducting a chi-square test or Fisher's exact test while continuous ones were compared by conducting a median two-sample test. Statistical analysis was done using SAS software. In both cohorts, all-cause-mortality was the primary outcome. Secondary outcomes included rate of ICU admission, rate of intubation, AKI, ARDS, ACI, thrombotic events, septic shock, effect on inflammatory markers, and duration of total hospital stay. Missing values were adjusted using f variance.

## Results

Of 270 hospitalized patients with COVID-19, 81 patients received TCZ. Fifty patients received one dose of TCZ and 31 received ≥ two doses. Median age (years) for cohort 1 was 63.5 and cohort 2 was 65.0 (p value=0.147). Cohort 1 was more likely to have male patients and the difference was statistically different (p value=0.0283). The patients in cohort 1 received TCZ on the third day (median) of hospitalization compared to cohort 2 who received the first dose on the second day of hospitalization. The median of lactate dehydrogenase (LDH), D-dimer, ferritin, and IL-6 at admission was higher in cohort 2 than cohort 1 (Table 1). The median values of CRPpeak, Ferritinpeak, and IL-6peak after receiving TCZ were lower in cohort 2 as compared to cohort 1. Of 81 patients, 23% received remdesivir, 28% received IV steroids, and 31% received convalescent plasma. There was no significant difference in both cohorts in concurrent treatment except TCZ. ICU admission rate, AKI, ARDS, ACI, thrombotic events, septic shock, and total hospital stay are mentioned in Table 2. All-cause-mortality in cohort 2 was higher (41.9%) than cohort 1 (36%). 

**Table 1:** Characteristics of cohort 1 and cohort 2. IQR, interquartile range; CAD, coronary artery disease; COPD, chronic obstructive pulmonary disease; OSA, obstructive sleep apnea; HTN, hypertension; DM, diabetes mellitus; CRP, C-reactive protein; LDH, lactate hydrogenase; FEU, fibrinogen equivalent units; TCZ, tocilizumab.

Parameters	All (n=81)	All TCZ	Cohort 1 (1 dose, n=50)	Cohort 2 (≥ 2 doses, n=31)	p-value
Baseline characteristics					
Age, years (IQR)	81	65.0 (55.0-71.0)	63.5 (54.0-69.0)	65.0 (59.0-78.0)	0.1474
Male, n (%)	56	56.0 (69.1)	39 (78.0)	17 (54.8)	0.0283
Female, n (%)	25	25 (30.86)	11 (22.0)	14 (45.2)	?
Comorbidities					
Heart failure-n, (%)		5 (6.2)	4 (8.0)	1 (3.2)	0.6443
CAD-n, (%)		9 (11.1)	7 (14.0)	2 (6.5)	0.4707
COPD-n, (%)		9 (11.1)	6 (12.0)	3 (9.7)	1.0000
OSA-n, (%)		2 (2.47)	0 (0)	2 (6.5)	0.1435
Obesity-n, (%)		7 (8.6)	4 (8.0)	3 (9.7)	1.0000
HTN-n, (%)		39 (48.2)	20 (40.0)	19 (61.3)	0.0623
DM-n, (%)		27 (33.3)	15 (30.0)	12 (38.7)	0.4190
CKD- n, (%)		13 (16.1)	6(12.0)	7 (22.6)	0.2281
Inflammatory markers at admission					
Median CRP, mg/L (IQR)	81	138.5 (85.6-198.6)	147.8 (103.3-211.0)	118.0 (74.4-172.3)	0.2942
Median LDH, IU/L (IQR)	81	452.0 (330.0-655.0)	433.0 (312.0-622.0)	583.0 (377.0-851.0)	0.0935
Median ferritin, ng/mL (IQR)	81	977.0 (399.0-1551.0)	963.0 (382.0-1625)	1130.0 (421.0-1391.0)	0.7534
Median IL-6, pg/mL (IQR)	63	108.4 (55.6-503.4)	107.8 (49.4-296.8)	163.7 (79.0-856.9)	0.3823
Median D-dimer, mg/L of FEU, (IQR)	81	1.1 (0.7-3.7)	1.0 (0.7-3.7)	1.6 (0.7-4.3)	0.4422
Peak inflammatory markers after receiving TCZ					
Median CRP, mg/L (IQR)	79	175.7 (135.6-275.8)	197.9 (139.3-326.0)	160.2 (105.8-209.5)	0.1303
Median LDH, IU/L (IQR)	79	664.0 (478.0-964.0)	580.5 (442.5-773.5)	851.0 (571.0-1045.0)	0.0091
Median ferritin, ng/mL (IQR)	79	1570.0 (931.0-3057.0)	1600.0 (872.5-3471.0)	1432.0(992.0-2785.0)	0.8894
Median IL-6, pg/mL (IQR)	20	3430.4 (659.5-16627.8)	3866.4 (1200.9-17210.0)	728.8 (578.3-5281.2)	0.1704
Median D-dimer, mg//L of FEU (IQR)	79	4.4 (1.6-4.4)	4.4 (1.3-4.4)	4.4 (2.5-4.4)	0.0971
TCZ first dose timing					
Before intubation-n, (%)	21	21 (26.0)	12 (24.0)	9 (30.0)	0.1965
After intubation-n, (%)	13	13 (16.0)	11 (22.0)	2 (6.0)	0.1965
Given in nonintubated patients-n, (%)	47	47 (58.0)	27 (54.0)	20 (64.0)	0.1965
Timing of first dose in hospital (days), median (IQR)	-	2.0 (2.0-4.0)	3.0 (2.0-5.0)	2.0 (1.0-4.0)	0.1622
Timing of first dose from symptoms onset (days), median (IQR)	-	9.0 (7.0-12.0)	9.0 (6.0-11.0)	9.0 (7.0-13.0)	0.1723
Timing of second dose in hospital (days), median (IQR)	-	-	-	4.0 (3.0-6.0)	-
Timing of second dose from symptom onset (days), median (IQR)	-	-	-	7.0 (3.0-10.0)	-
Other treatment					
Remdesivir-n, (%)		23 (28.4)	13 (26.0)	10 (32.3)	0.5438
Intravenous steroids-n, (%)		28 (34.6)	17 (34.0)	11 (35.5)	0.8914
Convalescent plasma-n, (%)		31 (38.3)	14 (28.0)	17 (54.8)	-

**Table 2:** Complications and outcomes in cohort 1 and cohort 2. ARDS, acute respiratory distress syndrome; AKI, acute kidney injury; LFT, liver function test.

Parameters	All (n=81)	All TCZ	Cohort 1 (1 Dose, n=50)	Cohort 2 (≥ 2 Doses, n=31)	p-value
Complications					
ARDS-n, (%)		48 (59.3)	28 (56.0)	20 (64.5)	0.4483
Acute cardiac injury-n, (%)		7 (8.6)	2 (4.0)	5 (16.1)	0.0999
AKI-n, (%)		28 (34.6)	15 (30.0)	13 (41.9)	0.2723
Septic shock-n, (%)		25 (30.9)	15 (30.0)	10 (32.3)	0.8307
Raise in LFT after IL-6 inhibitor-n, (%)		44 (54.3)	27 (54.0)	17 (54.8)	0.9413
Superimposed blood infections-n, (%)		15 (18.5)	9 (18.0)	6 (19.4)	0.8787
Thrombotic events-n, (%)		3 (3.7)	2 (4.0)	1 (3.2)	1.0000
Final outcomes					
ICU level of care-n, %		43 (53.1)	26 (52.0)	17 (54.8)	0.8035
Intubated-n, (%)		33 (40.7)	23 (46.0)	11 (35.5)	0.2212
Discharged alive from hospital-n, (%)		50 (61.7)	32 (64.0)	18 (58.1)	0.5932
Dead-n, (%)		32 (39.5)	18 (36.0)	13 (41.9)	0.4123
Extubated-n, (%)		7 (21.2)	5 (21.7)	2 (20.0)	1.0000
Median duration of hospital stay, days (IQR)		12.0 (8.0-24.0)	10.5 (7.0-24.0)	13.0 (10.0-22.0)	0.2058
Median duration of illness onset to discharge/death, days (IQR)		18.0 (13.0-31.0)	16.5 (12.0-34.0)	20.0 (15.0-31.0)	0.3133

## Discussion

Tocilizumab is a recombinant humanized anti-human IL-6 receptor monoclonal antibody, which is currently FDA approved for rheumatoid arthritis-related inflammation, giant cell arteritis, systemic and polyarticular juvenile idiopathic arthritis, and CAR-T therapy-induced cytokine release syndrome. In our study, TCZ was initially administered as a single dose of 400-800 mg (based on the weight) among the hospitalized COVID-19 patients. Additional doses were given as a result of worsening respiratory status as measured by increasing O2 requirements. These additional doses were given at least 24 hours apart (Table 1). The two cohorts did not have significant differences in baseline characteristics including age, comorbidities [coronary artery disease (CAD), diabetes mellitus (DM), chronic obstructive pulmonary disease (COPD), and obesity), other treatments including steroids, remdesivir, and convalescent plasma. This suggests that both cohorts had relatively comparable risk factors for disease severity. The median of LDH, D-dimer, ferritin, and IL-6 at admission was higher in cohort 2, however, p-values for inflammatory markers were not statistically significant. This can correlate with disease severity and may suggest cohort 2 had a sicker patient population than cohort 1. This may indicate why the first dose was given earlier (median of two days) followed by additional doses in cohort 2. All-cause-mortality in cohort 2 remained higher (41.9%) suggesting that there was no additional benefit of multiple doses of TCZ to prevent the primary outcome. In addition, multiple doses of TCZ did not change any other secondary outcome (ICU admission, AKI, ARDS, ACI, thrombotic events, septic shock, and total hospital stay). Lower median values of Ferritinpeak, IL-6peak, and CRPpeak in cohort 2 suggest that multiple doses of TCZ might have played a role to mitigate the cytokine storm, however, a statistically significant difference was not observed. A recent RCT indicated that TCZ might decrease the risk of mechanical ventilation (MV), as seen in our study; cohort 2 were less likely to be intubated (p-value 0.22) [2]. IL-6 plays a role in defense against infections and increases the rate of secondary infections when used chronically [6]. Extended hospital stay is seen commonly in severe COVID-19 patients which is often complicated by superimposed secondary infections. It occurred in 18.5% of patients in our study group, with no statistical significance between two cohorts. Our data indicate that there was no additional benefit of multiple doses of TCZ in hospitalized COVID-19 patients. The major limitations of our study include retrospective study design and single-center data. Cohort 2 had a sicker patient population compared to cohort 1. We speculate that the role of multiple doses should be investigated further in prospective randomized optimal dose-finding trials with a larger sample size for definitive conclusions.

## Conclusions

Our study revealed that multiple doses of TCZ did not decrease the all-cause-mortality, or secondary outcomes (ICU admission, AKI, ARDS, ACI, thrombotic events, septic shock, and total hospital stay) in cohort 2 compared to cohort 1. 

## References

[1] Francesca C, Chiovato L, Croce L, Magri F, Rotondi M (2020). The cytokine storm in COVID- 19: an overview of the involvement of the chemokine/chemokine-receptor system. Cytokine Growth Factor Rev.

[2] Hermine O, Mariette X, Tharaux P-L (2020). Effect of tocilizumab vs usual care in adults hospitalized with COVID-19 and moderate or severe pneumonia. JAMA Intern Med.

[3] Sinhaa P, Mostaghimb A, Bielick CG (2020). Early administration of interleukin-6 inhibitors for patients with severe COVID-19 disease is associated with decreased intubation, reduced mortality, and increased discharge. Int J Infect Dis.

[4] Lee DW, Gardner R, Porter DL (2015). Current concepts in the diagnosis and management of cytokine release syndrome. Blood. 2014;124(2): 188-195. Blood.

[5] Le RQ, Li L, Yuan W (2018). FDA approval summary: tocilizumab for treatment of chimeric antigen receptor T cell‐induced severe or life‐threatening cytokine release syndrome. Oncologist.

[6] Pawar A, Desai RJ, Solomon DH (2019). Risk of serious infections in tocilizumab versus other biologic drugs in patients with rheumatoid arthritis: a multidatabase cohort study. Ann Rheum Dis.

